# Substantial Changes in Selected Volatile Organic Compounds (VOCs) and Associations with Health Risk Assessments in Industrial Areas during the COVID-19 Pandemic

**DOI:** 10.3390/toxics11020165

**Published:** 2023-02-09

**Authors:** Bhupendra Pratap Singh, Sayed Sartaj Sohrab, Mohammad Athar, Thamir A. Alandijany, Saumya Kumari, Arathi Nair, Sweety Kumari, Kriti Mehra, Khyati Chowdhary, Shakilur Rahman, Esam Ibraheem Azhar

**Affiliations:** 1 Department of Environmental Studies, Deshbadhu College, University of Delhi, New Delhi 110019, India; 2 Delhi School of Climate Change and Sustainability (Institute of Eminence), University of Delhi, New Delhi 110007, India; 3 Special Infectious Agents Unit, King Fahd Medical Research Center, King Abdulaziz University, Jeddah 21589, Saudi Arabia; 4 Department of Medical Laboratory Sciences, Faculty of Applied Medical Sciences, King Abdulaziz University, Jeddah 21589, Saudi Arabia; 5 Science and Technology Unit, Umm Al-Qura University, Makkah 21955, Saudi Arabia; 6 Department of Medical Genetics, Faculty of Medicine, Umm Al-Qura University, Makkah 21955, Saudi Arabia; 7 Department of Zoology, Deshbandhu College, University of Delhi, New Delhi 110019, India; 8 Department of Life Science, Deshbadhu College, University of Delhi, New Delhi 110019, India; 9 Department of Medical Elementology and Toxicology, School of Chemical and Life Sciences, Jamia Hamdard, New Delhi 110019, India

**Keywords:** TVOCs, pandemic, T/B ratio, meteorological parameters, LCR

## Abstract

During the COVID-19 pandemic, governments in many countries worldwide, including India, imposed several restriction measures, including lockdowns, to prevent the spread of the infection. COVID-19 lockdowns led to a reduction in gaseous and particulate pollutants in ambient air. In the present study, we investigated the substantial changes in selected volatile organic compounds (VOCs) after the outbreak of the coronavirus pandemic and associations with health risk assessments in industrial areas. VOC data from 1 January 2019 to 31 December 2021 were collected from the Central Pollution Control Board (CPCB) website, to identify percentage changes in VOC levels before, during, and after COVID-19. The mean TVOC levels at all monitoring stations were 47.22 ± 30.15, 37.19 ± 37.19, and 32.81 ± 32.81 µg/m^3^ for 2019, 2020, and 2021, respectively. As a result, the TVOC levels gradually declined in consecutive years due to the pandemic in India. The mean TVOC levels at all monitoring stations declined from 9 to 61% during the pandemic period as compared with the pre-pandemic period. In the current study, the T/B ratio values ranged from 2.16 (PG) to 26.38 (NL), which indicated that the major pollutant contributors were traffic and non-traffic sources during the pre-pandemic period. The present findings indicated that TVOC levels had positive but low correlations with SR, BP, RF, and WD, with correlation coefficients (r) of 0.034, 0.118, 0.012, and 0.007, respectively, whereas negative correlations were observed with AT and WS, with correlation coefficients (r) of −0.168 and −0.150, respectively. The lifetime cancer risk (LCR) value for benzene was reported to be higher in children, followed by females and males, for the pre-pandemic, pandemic, and post-pandemic periods. A nationwide scale-up of this study’s findings might be useful in formulating future air pollution reduction policies associated with a reduction in health risk factors. Furthermore, the present study provides baseline data for future studies on the impacts of anthropogenic activities on the air quality of a region.

## 1. Introduction

Coronavirus disease (COVID-19) is a disease caused by severe acute respiratory syndrome coronavirus 2 (SARS-CoV-2), and was first reported in Wuhan, China, in late December 2019 [1]. It rapidly spread across the world in a short time span, and the World Health Organization declared it a pandemic on 11 March 2020 [2]. The WHO and other agencies reported that, as of 5 January 2023, COVID-19 infected more than 66 million people, with more than 6.7 million deaths globally (including in India) [3]. The United States of America (USA) was the most adversely affected country, followed by India, the second most afflicted country in the world, with more than 10 million COVID cases and more than 1.1 million deaths reported as of 5 January 2023 [4]. 

Few studies have reported a relationship between air pollution and infectious disease transmission [5,6]. Several early pieces of evidence suggest that the link between prolonged exposure to air pollution and the impact of COVID-19 might increase the probability of severe outcomes [7,8,9,10]. Many researchers suggested that air pollutants may influence the severity of COVID-19 associated with respiratory infection [11], cardiovascular disease [12], as well as morbidity and mortality [13,14]. Among the air pollutants, volatile organic compounds (VOCs) are considered principal components and are often designated as specific hazardous or toxic air pollutants [15,16]. These VOCs also play a crucial role in forming tropospheric ozone and secondary pollutants through photochemical smog [17,18,19,20]. In many cities worldwide, significant reductions in atmospheric pollutant concentrations were observed during the lockdown periods of COVID-19 due to the complete or partial closures of industries, as well as transport and construction works [21,22,23]. It is very difficult to assess the air quality with respect to the contributions of different pollutants, and changes in individual pollutant levels are difficult to link to overall air pollution; therefore, it is difficult to compare their impacts on human health associated with concentrations of different pollutants [24,25]. Volatile organic compounds (VOCs) and nitrogen oxides (NOx) are the two main forerunners to tropospheric O_3_ formation, with complex chemical mechanisms found in them [26,27]. The photochemical process depends upon the VOC to NOx ratio in the atmosphere, which has a pivotal role in O_3_ formation [28].

Up to 60% of non-methane VOCs (NMVOCs) released into the atmosphere are BTEX [28]), and changes in BTEX ratios can be used as effective tools for investigating the causes of different photochemical processes that occur in the environment [17,29]. Traffic-related VOCs and VOCs released by industries as well as changes in VOC levels from many individual sources have been investigated to estimate the impact of the COVID-19 lockdown on the environment. Additionally, in some particular compounds, such as benzene, toluene, ethyl benzene, and xylene (represented by the acronym BTEX), some harmful effects of VOCs have been shown via short- and long-term adverse health effects. Furthermore, VOCs that are released into the ambient environment from various sources, such as oil and gas, play very crucial and important roles in petrochemical activities [16]. BTEX compounds are considered to be the main components of gasoline, and, due to their high evaporation rate, they can enter the ambient air environment from outer exhausts, vehicle carburetor engines, and petroleum product distribution stations [30]. Emission intensities of pollution sources and meteorological conditions play important roles in varying VOC levels, while in the present scenario, meteorological conditions significantly influence the chemical transformations involved in the production of O_3_ concentrations [27]. During COVID-19, there were many studies under highly unusual conditions of partial or total internment and, therefore, vital information could be acquired for designing policies and strategies to prevent and control air pollution through evaluations of the effects of reduced emission sources on the local urban air quality [21,22,23,31]. 

Changes in atmospheric pollutants during the COVID-19 lockdown periods have been widely investigated; researchers have reported significant reductions in nitrogen dioxide (NO_2_), particulate matter (PM), and carbon monoxide (CO) levels in many different cities across the world [10,23,24]. Subali et al. (2021) revealed a potential VOC-based breath analysis associated with high sensitivity and promising specificity for COVID-19 screening [32]. Another study conducted in Maharashtra (India) reported that total VOC levels decreased during the lockdown periods in the corresponding year, 2019 [33]. However, due to shallower boundary layer depths, higher concentrations of aromatic volatile organic compounds (VOCs) and CO were found in the wintertime and transported from the polluted Indo-Gangetic Plain region. Relatively high loadings of benzene (~30%), toluene (45%), and CO (32%), respectively, were observed in vehicle exhaust by using the positive matrix factorization analysis method [34].

According to Ghaffari et al. (2021), the most toxic BTEX compound is benzene, which has been categorized as a Group 1 and class A human carcinogenic by the International Agency for Research on Cancer (IARC) and the United States Environmental Protection Agency (U.S. EPA), respectively [35]. Several studies have reported that individual VOCs are significantly associated with the adverse effects of cardiovascular and respiratory diseases [36,37] asthma [38], and chronic obstructive pulmonary disorder [39], and could possibly increase the chances of leukemia and aplastic [40]. Several studies have claimed that a considerably high concentration of benzene was associated with a high cancer risk for lifetime exposure in an ambient environment [41,42]. 

Changes in VOC levels due to particle and gaseous contaminants, in particular, have received more attention and several studies of various cities in India have revealed that the air quality improved significantly during the pandemic period [23,24,25]. Nevertheless, only a few studies have discussed links between BTEX compounds and health. In addition, there is a paucity of thorough research on BTEX compounds as well as the pandemic’s health risks, and BTEX compounds during lockdown periods in North India have not been examined in any prior study. The key aims of the present study are: (i) to evaluate the spatiotemporal variations in TVOC levels, (ii) to identify the sources of BTEX, and (iii) to calculate the health risks associated with BTEX across various age groups.

## 2. Methodology

### Study Area

**Figure 1:**
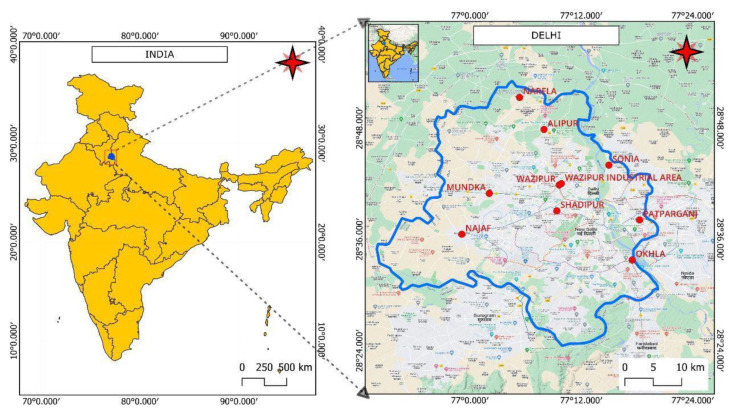
Sampling stations for all monitoring sites at Delhi.

The National Capital Territory of Delhi, India, has coordinates of 28.70° N and 77.10° E. It is situated on the Indo-Gangetic Plain of the northern region of India [24,25]. One report suggests that the National Capital Territory has subtropical and semi-arid climatic conditions. The area experiences all seasons, including summer, monsoon, and winter from April to June, from July to October, and from November to February, respectively. The climate of Delhi is humid and is greatly impacted by the annual monsoon. The average temperature from May to June is 35–40 °C, and from November to February it is 5–7 °C. The humidity is mostly felt during the months of July and August. Usually, there is a northeastern breeze in Delhi, but during the late summers, it is replaced by a southeastern wind. The sampling locations for all monitoring sites are presented in Figure 1. 

Delhi is among the most polluted cities in India according to the index of global pollution [43]. Delhi is also one of the most populated cities in India, with 13.4 million registered vehicles on the roads [44]. In addition, Delhi’s Metropolitan area has a huge number of public and private transportation vehicles compared with other Indian cities. Therefore, industrial areas play crucial roles in enhancing the level of pollution. In addition, automobiles, construction, and other anthropogenic activities are important key factors. All of these factors lead to the emissions of various compounds, including carcinogenic volatile organic compounds (VOCs). In the present study, selected monitoring stations in Delhi are presented in Table 1.

## 3. Data and Sources

In the present study, hourly and daily data of volatile organic compounds, especially benzene, toluene, ethylene, and xylene (BTEX), were collected from the Central Pollution Control Board (CPCB) website (https://app.cpcbccr.com/ccr/#/caaqm-dashboard-all/caaqm-landing, accessed on 5 January 2022). The measurements and technical specifications of the instruments can be found elsewhere [45].

Several previous studies have reported that the CPCB provides data quality assurance and quality control (QA/QC) programs and detection limits of each BTEX compound through rigorous sampling, analysis, and calibration procedures [35].

In the present study, data were procured from 1 January 2019 to 31 December 2021, to identify percentage changes in the VOC levels (https://app.cpcbccr.com/ccr/#/caaqm-dashboard-all/caaqm-landing, accessed on 5 January 2023). The data were procured in three time periods before, during, and after COVID-19. To examine the relative and temporal changes in VOC levels in the ambient atmosphere, the time period between 1 January 2019 and 31 December 2019 represented the pre-pandemic period, the time period between 1 January 2020 and 31 December 2020 represented, the pandemic period, and the time period between 1 January 2021, and 31 December 2021, represented the post-pandemic period. All 10 industrial air quality monitoring stations in Delhi that were selected for this study, with their latitudes, longitudes, and population census data, are presented in Table 1. The monitoring stations are Alipur (AL), Bawana (BW), Mundka (MD), Najafgarh (NG), Narela (NL), Okhla (OKH), Patparganj (PG), Shadipur (SP), Sonia Vihar (SON), and Wazirpur (WA). Meteorological parameters, such as solar radiation (SR in kWh/m^2^), pressure (BP in kg/ms^2^), atmospheric temperature (AT in Celsius), rainfall (RF in mm), wind speed (WS in km/h), and wind direction (WD in degree/cardinal direction) were observed on an hourly basis at all 10 monitoring sites.

## 4. Human Health Risk Assessment

A human health risk assessment (HHRA) can be performed to assess the nature and probability of different pollutants in a population based on acute and chronic exposure.

### 4.1. Hazard Identification

The pollutants that cause major impacts on human health are considered hazardous. In this current study, VOCs such as BTEX are hazardous to human health and can cause cancer.

### 4.2. Exposure Assessment

An exposure assessment (EA) was performed to examine the duration and magnitude of the pollutants based on different parameters. In the present study, inhalation was the major route of exposure for the identified pollutants. We estimated the daily and annual readings of normal and acute exposure periods for different age groups, namely males (70 years), females (60 years), and children (36 years) [46]. The values of the parameters used in the health risk assessment model are presented in Table 2.

(1) $$\mathrm{EC}\left(\frac{µ}{m^{3}}\right)=\frac{\mathrm{CA}\left(\frac{µ}{m^{3}}\right)\times \mathrm{ET}\left(\frac{h}{\mathrm{day}}\right)\times \mathrm{EFyear}\times \mathrm{ED}\left(\frac{\mathrm{day}}{\mathrm{year}}\right)}{\mathrm{AT}\;\left(\mathrm{year}\right)\times 365\left(\frac{\mathrm{day}}{\mathrm{year}}\right)\times 24\left(\frac{h}{\mathrm{day}}\right)}$$

(2) $$\mathrm{EDI}\left(\frac{\mathrm{mg}}{\mathrm{kg}.\mathrm{day}}\right)=\frac{\mathrm{CA}\left(\frac{µ}{m^{3}}\right)\times \left(\frac{1}{1000}\right)\left(\frac{\mathrm{mg}}{µg}\right)\times \mathrm{IR}\left(\frac{m3}{\mathrm{day}}\right)\times \mathrm{ET}\left(\frac{h}{\mathrm{day}}\right)\times \mathrm{EFyear}\times \mathrm{ED}\left(\frac{\mathrm{day}}{\mathrm{year}}\right)}{\mathrm{AT}\;\left(\mathrm{year}\right)\times 365\left(\frac{\mathrm{day}}{\mathrm{year}}\right)\times 24\left(\frac{h}{\mathrm{day}}\right)\times \mathrm{BW}\left(\mathrm{kg}\right)}$$

(3) $$\mathrm{HQ}=\frac{\mathrm{EC}}{\mathrm{RfC}}$$

(4) ILCR=CDI×SF

where EC (µg/m^3^) represents the exposure concentration, defined as the number of TVOCs present per cubic meter; CA (µg/m^3^) = VOC, the average concentrations of benzene, toluene, ethylene, and xylene; ET (h/d) is the exposure time, the total time duration per day in which exposure to TVOCs takes place; EF (d/y) represents exposure frequency, defined as the number of exposures taking place in a day;

ED (y) represents the exposed length of working, the difference of the average age of exposure and the average age at the beginning; AT (h) is the average exposure time, during the carcinogenic assessment, the average lifetime (per capita life expectancy × 365 d/y × 24 h/d) was adopted, and during the non-carcinogenic assessment, the average period of exposure cycle (ED × 365 d/y × 24 h/d) was adopted; HQ (µg/m^3^) is the hazard quotient, the ratio of exposure to chemicals and the measure at which no defined results can occur; RfC is the reference concentration of inhalation toxicity, which refers to continuous exposure to the human population without any cancerous health risks; SF (kg d mg ^−1^) represents the carcinogenic slope factor, defined as an upper bound, approximating a 95% confidence limit in the escalated cancer crisis from the lifetime exposure to a chemical [23]; ILCR (incremental lifetime cancer risk) refers to increasing the chances of any person having cancer due to exposure to a pollutant during his/her lifetime.

## 5. Results and Discussion

### 5.1. Total VOC Levels for 2019–2021

**Figure 2:**
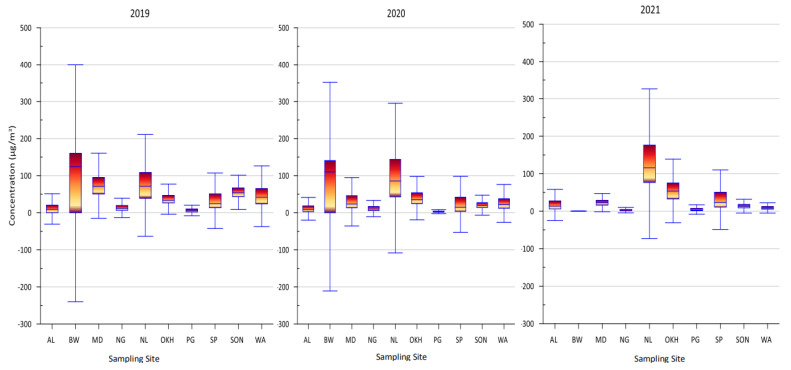
Box plot for TVOCs for different monitoring stations in Delhi for the years 2019, 2020, and 2021.

The current study focused on establishing the significant changes in air pollutants in different industrial zones, especially the total volatile organic compound (TVOC) levels, from 2019 to 2021, in Delhi, India. At all monitoring stations, the mean TVOC levels were 47.22 ± 30.15, 37.19 ± 37.19, and 32.81 ± 32.81 µg/m^3^ for 2019, 2020, and 2021, respectively (Figure 2). The results show that the TVOC levels gradually deteriorated over successive years due to the pandemic in India. The main aspects behind the significant decrease in TVOC levels during the lockdown were complete and partial restrictions on transport, industrial activities, and marketplace openings. The annual mean TVOC levels at all monitoring stations ranged from 6.70 ± 4.71 to 103.86 ± 80.37, from 3.65 ± 7.36 to 97.57 ± 68.39, and from 5.21 ± 5.12 to 128.56 ± 74.43 µg/m^3^, for 2019, 2020, and 2021, respectively. The trend of annual mean TVOC levels was observed to be BW > MD > NL > SON > WA > OKH > SP > AL > NG > PG for 2019, whereas the trend was NL > BW > OKH > SP > MD > SON > NG > AL > PG for 2020, and NL > OKH > SP > MD > BW > AL SON > WA > PG > NG for 2021.

The maximum TVOC levels were 347.27 µg/m^3^ (BW), 408.91 µg/m^3^ (NL), and 467.30 µg/m^3^ (NL) for 2019, 2020, and 2021, respectively. The maximum TVOC levels at all stations varied from 23.40 µg/m^3^ (PG) to 347.27 µg/m^3^ (BW), from 75.06 µg/m^3^ (SON) to 408.91 µg/m^3^ (NL), and from 37.18 µg/m^3^ (PG) to 467.30 µg/m^3^ (NL) for 2019, 2020, and 2021, respectively. 

### 5.2. Identification of VOC Characteristic Pollutants for 2019

The BTEX concentrations for 2019–2021 at all selected monitoring regions are presented in Figure 2. The mean values for individual VOCs were 3.48 ± 2.43, 48.33 ± 22.86, 3.68 ± 12.29, and 3.7 ± 3.64 µg/m^3^ for benzene, toluene, eth-benzene, and mp-xylene, respectively, for 2019. The average concentration values for benzene at all monitoring stations varied from 1.26 (AL) to 5.43 (SP) µg/m^3^, whereas, for toluene, the average concentration values varied from 4.58 (PG) to 74.22 (MD) µg/m^3^. The trend of the mean benzene value was observed to be SP > OKH > MD > SON > WA > NL > PG > NG > AL; the trend of the mean toluene value was NL > MD > SON > WA > OKH > SP > AL > NG > PG.

### 5.3. Identification of VOC Characteristic Pollutants for 2020

The mean values of benzene, toluene, eth-benzene, and mp-xylene at all monitoring stations were calculated to be 12.85 ± 9.42, 30.42 ± 19.06, 4.06 ± 7.06, and 8.60 ± 13.71 µg/m^3^, respectively, for 2020–2021. The standard deviation value was high, indicating large variations in emission sources at various monitoring stations. The average benzene values at the monitoring stations ranged from 1.47 (AL) to 98.42 (NL) µg/m^3^, whereas the average toluene values ranged from 2.39 (NG) to 145.22 (BW) µg/m^3^, respectively. The maximum mean values among all monitoring stations were reported at Narela (benzene) and Bawana (toluene) monitoring stations, which are hubs of industrial zones in Delhi. Most plastic industries in BW operated during the pandemic period due to their association with the production of sanitizing bottles. These industries might have contributed more benzene and toluene compound emissions. Considering all of the selected monitoring stations, the trend for the average benzene values was NL > NG > SP > WA > SON > OKH > MD > AL > PG > BW, and the trend for the average toluene values was BW > OKH > MD > WA > SP > SON > AL > PG > NL > NG.

### 5.4. Identification of VOC Characteristic Pollutants for 2021

The mean values for individual VOCs at all monitoring stations were 2.89 ± 2.66, 43.01 ± 22.26, 505 ± 5.01, and 6.23 ± 8.14 µg/m^3^ for benzene, toluene, eth-benzene, and mp-xylene, respectively, for 2021–2022. The average values for benzene at the monitoring stations ranged from 5.26 (SP) to 0.55 (PG), whereas the average values for toluene ranged between 164.91 (BW) and 0.91 (NG) µg/m^3^. Considering all of the selected monitoring stations, the trend of average benzene values was SP > OKH > MD > SON > NL > WA > AL NG > PG > PG, and the trend for average toluene values was BW > NL > OKH > SP > MD > AL > SON > WA PG > NG. 

### 5.5. Comparative Analysis of Pre-Pandemic, Pandemic, and Post-Pandemic Periods

**Figure 3:**
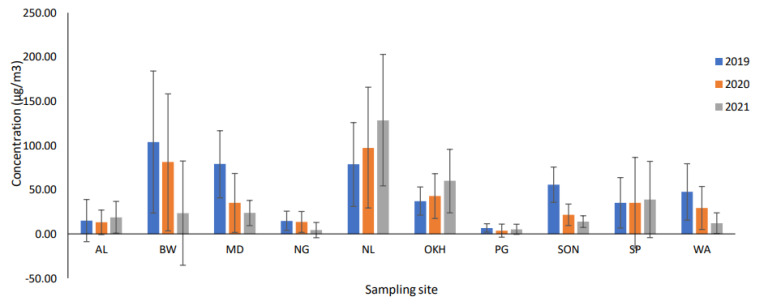
A comparative analysis of TVOCs at all sampling sites for the years 2019, 2020, and 2021.

The average TVOC values at all of the monitoring stations declined from 9 to 61% during the pandemic period compared with those of the pre-pandemic period. The highest decline was observed at the SON monitoring station (−61%) and the lowest decline was at the NG monitoring station (−9%); the reason could be that the SON monitoring station was observed to restrict measures during the pandemic period, which caused significant changes in TVOC values compared with the pre-pandemic period (Figure 3). However, the NG monitoring station is considered to be India’s second most pollutant cluster, with air and water in the critical category. On the one hand, most industrial activities were performed during the pandemic period, and there were insignificant changes in TVOC values compared with those of the pre-pandemic period. On the other hand, increased TVOC values were reported at OKH (24%) and NL (15%) monitoring stations during the pandemic period. The location of the OKH monitoring station is considered to be an industrial zone (waste-to-energy plant) where municipal solid wastes (generated from households) are used as fuel, which continued during the pandemic period [24,25]. Therefore, the increase in the level of VOCs reported by the OKH monitoring station was attributed to waste burning. Several previous studies have reported that higher source emissions could be attributed to local source emissions from burning waste and construction activities near a monitoring station [47]. 

The average TVOC values continued to decline even during the post-pandemic period due to restricted measures in a few of the monitoring stations. The government declared restricted measures for the post-pandemic period, under which schools, colleges, cinemas, and gyms were to remain closed, shops dealing in non-essential items were opened only on an odd–even basis, and metro trains and buses in the city ran at 50% seating capacity.

The decreases in TVOC levels varied from −77% (BW) to −22% (PG), whereas there were significant increases in TVOC levels with changes at 64%, 62%, and 11% at the NL, OKH, and SP monitoring stations. The highest increase in TVOC levels was reported at the NL monitoring station because the NL monitoring station is located near plastic industries for making shoe soles and other plastic goods, such as Rexine, adhesives, and other highly inflammable items, which could be a significant source of emissions during the reopening of industrial activities. Similarly, the OKH monitoring station witnessed a further increase in the amount of waste generated from domestic and industrial sectors, from BTEX pollutant sources that included plastics, paints, resins, rubber, adhesives, lubricants, and detergents [30].

A study in Maharashtra reported that TVOC levels declined by 84% during the lockdown period as compared with those of the previous year [23]. The average TOVC value at the PG monitoring station declined by 46% and 22% during the pandemic and post-pandemic periods, respectively, as compared with that of the pre-pandemic period. Various industrial sectors, such as paper, scraps of leather, and polythene, were located near the PG monitoring station, which attributed to source emissions. The decline in the average TVOC levels was higher during the pandemic (46%) compared with the post-pandemic period due to the reopening of these industrial activities. Additionally, the PG monitoring station is located near the Ghazipur landfill site, which further contributed to higher VOC levels in this monitoring station.

### 5.6. Source Identification

Identification and estimation of VOC emission sources can be assessed using diagnostic ratios. The toluene/benzene ratio can be used to evaluate the impact of traffic and non-traffic sources [23]. The ratio of toluene/benzene (T/B) is frequently used to inspect the relative importance of vehicular exhaust, industrial emissions, and combustion sources and to provide crucial insight into the vicinity of vehicular discharge sources and photochemically aged air masses [48]. A T/B ratio that is less than 2 indicates that vehicular emissions have a significant influence on aromatic VOC emissions. Several studies have reported that T/B ratios close to or more than 2 refer to non-traffic sources, and ratios higher than 10 indicate industrial activity as a considerable factor [23,48,49].

**Figure 4:**
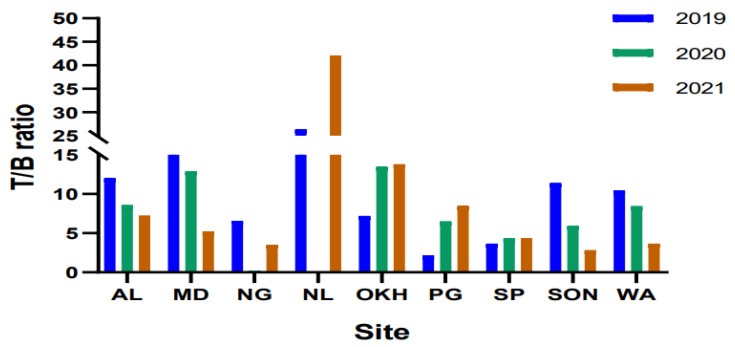
T/B ratio at all industrial monitoring stations for years 2019, 2020, and 2021.

In the current study, the T/B ratio values ranged from 2.16 (PG) to 26.38 (NL), indicating that the major VOC contributors were the traffic and non-traffic sources during the pre-pandemic period. The calculated T/B ratios at some monitoring stations, such as the WZ, SON, AL, MD, and NL monitoring stations, were reported to be 10.44, 11.39, 12.00, 16.49, and 26.38, respectively, indicating the activities of industries and factories were the primary causes of VOC emissions (Figure 4). During 2020, the T/B ratios ranged from 0.03 (NL) to 13.47 (OKH), indicating that the major VOC contributors were traffic and non-traffic sources. Narela and Najafgarh had ratios of 0.03 and 0.21, respectively, stipulating that vehicular emissions were the main sources of VOC emissions. The T/B ratio during 2021 ranged from 3.54 (NG) to 42.10 (NL), where high ratios indicated non-traffic sources and much higher ratios indicated industries, factories, and petrol pumps as the predominant VOC contributors. The calculated T/B ratios at OKH and NL monitoring stations were 13.81 and 42.10, respectively, indicating that the activities of industries and factories were the predominant sources of VOC emissions.

### 5.7. Correlations among the Monitoring Stations

Correlations among VOC levels can help one to understand the source of origin of the different constituents. If the correlation between different pollutants is similar, it depicts that their source of origin might be the same. The correlation value was found by using the Pearson correlation coefficient for the mean VOC levels on a daily basis. These correlation data were classified into various categories depending on the coefficient value (from −1 to 1). A coefficient value from 0.8 to 1.0 indicated a very strong correlation, a coefficient value from 0.6 to 0.8 indicated a strong correlation, a coefficient value from 0.4 to 0.6 indicated a moderate correlation, a coefficient value from 0.2 to 0.4 indicated a weak correlation, and a coefficient value from 0 to 0.2 indicated irrelevant data [50]. In the present study, a very strong positive correlation was observed between the NG and MD monitoring stations (0.734) during the pre-pandemic period, whereas other monitoring stations reported moderate to low correlations. In a similar study in Maharashtra, the authors reported a significantly strong correlation between the Thane and Bandra monitoring stations (0.73) in the pre-lockdown period [19]. The correlations among the different monitoring stations are presented in Table 2, Table 3 and Table 4.

During the pandemic period, there was a strong positive correlation between the SP and AL monitoring stations (0.736) and between the SP and OKH monitoring stations (0.619). For the post-pandemic period, strong positive correlations were shown for the BW and WA (0.813), BW and NG (0.646), PG and OKH (0.725), SP and AL (0.654), OKH and SON (0.612), SON and SP (0.617), and WZ and NG (0.648) monitoring stations. Apart from this, various monitoring stations showed moderate correlations, such as WA and AL (0.572), and WA and SP (0.587) monitoring stations. 

### 5.8. Correlations between the TVOC Levels and Meteorological Parameters

In the present study, we investigated the correlations between total VOC (TVOC) levels and meteorological parameters during the period from 2019 to 2021. Table 5 shows the correlation statistics of the TVOC significant levels (*p* = 0.05) with solar radiation, pressure, atmospheric temperature, rainfall, wind speed, and wind direction. 

Our results indicated that TVOC level had positive but low correlations with SR, BP, RF, and WD with correlation coefficients (r) of 0.034, 0.118, 0.012, and 0.007, respectively, whereas negative correlations were observed with AT and WS with correlation coefficients (r) of −0.168 and −0.150, respectively. These observations indicated that VOC levels were lower during high AT and WS, possibly due to photodegradation and wind dispersion, which played crucial roles in the VOC levels. Similarly, RF showed a strong negative correlation with BP (−0.992) and a lesser correlation with AT (−0.070). A similar study in Delhi reported variations in pollutant concentrations associated with meteorological parameters [51,52,53].

### 5.9. Health Risk Assessment

#### Hazard Quotient (HQ)

**Figure 5:**
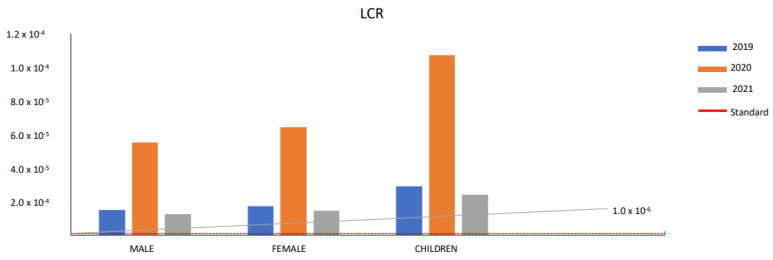
Lifetime cancer risk value for different gender for the years 2019, 2020, and 2021.

The hazard quotient (HQ) defines the ratio of the exposure concentration for the specific VOC species to an acute reference concentration (RfC) of non-carcinogenic compounds [54,55]. An HQ value of less than 1 indicates a minor or insignificant non-carcinogenic effect, whereas higher values indicate greater non-carcinogenic risks resulting in significant adverse effects on human health [56,57,58]. The current study estimated the total HQ values for benzene for the years 2019, 2020, and 2021 to be 0.11, 0.43, and 0.09 for males, 0.13, 0.51, and 0.11 for females, and 0.23, 0.85, and 0.19 for children at all industrial sites, respectively. All HQ values were reported to be below 1, indicating negligible human health risks [59]. A similar study conducted in industrial regions reported the HQ values to be less than 0.1 in Tehran, Iran [59] and Rayong Province, East Thailand [58]. According to Baberi et al. (2022), during the lockdown, people spent more than 80% of their schedules in enclosed areas that were associated with hazardous pollutants (benzene), which helped lower the load of disease and thereby reduced national healthcare costs [60]. For the pre-pandemic, pandemic, and post-pandemic periods, for all monitoring regions, in the present study, we estimated the LCR values for benzene in all age groups for males, females, and children, as shown in Figure 5.

For males, the total LCR values for benzene at all monitoring stations were calculated as 1.49 × 10^−5^, 5.51 x 10^−5^, and 1.24 × 10^−5^, whereas the values for toluene were calculated as 1.5 × 10^−4^, 1.57 × 10^−4^, and 1.5 × 10^−4^, for 2019, 2020, and 2021, respectively. The LCR values for benzene at all monitoring stations varied from 5.41 × 10^−6^ to 2.32 × 10^−5^, from 1.24 × 10^−6^ to 4.22 × 10^−4^, and from 2.36 × 10^−6^ to 2.26 × 10^−5^ for 2019, 2020, and 2021, respectively. The LCR values for benzene at all monitoring stations were estimated as the lowest for AL and the highest for SP in 2019, while in 2020 they were the lowest for BW and the highest for NL, and in 2021 they were the lowest for PG and the highest for SP. The results indicated that LCR values were higher during the pandemic period than those in the pre- and post-pandemic periods. For benzene, some monitoring stations had LCR values that exceeded the standard LCR value as prescribed by the CPCB (1.0 × 10^−6^), such as values of 1.23 × 10^−5^, 1.78 × 10^−5^, 1.92 × 10^−5^, 1.93 × 10^−5^, 1.93 × 10^−5^, and 2.33 × 10^−5^ for NL, WZ, SON, MD, OKH, and SP, respectively, in 2019; 1.08 × 10^−5^, 1.27 × 10^−5^, 1.35 × 10^−5^, 1.35 × 10^−5^, and 1.99 × 10^−5^ for MD, OKH, SON, WZ, and SP, respectively, in 2020; 1.11 × 10^−5^, 1.28 × 10^−5^, 1.55 × 10^−5^, 1.69 × 10^−5^, 1.73 × 10^−5^, and 2.26 × 10^−5^ for WZ, NL, SON, MD, OKH, and SP, respectively, in 2021.

For females, the totals of LCR values for benzene at all monitoring stations were calculated to be 1.73 × 10^−5^, 6.42 × 10^−5^, and 1.44 × 10^−5^, whereas the values for toluene were calculated to be 1.82 × 10^−4^, 1.82 × 10^−4^, and 1.82 × 10^−4^ in 2019, 2020, and 2021, respectively. The LCR values for benzene at all of the monitoring stations varied from 6.31 × 10^−6^ to 2.71 × 10^−5^, from 1.45 × 10^−6^ to 4.92 × 10^−4^, and from 2.76 × 10^−6^ to 2.63 × 10^−5^ for 2019, 2020, and 2021, respectively. The LCR values for benzene, at all of the monitoring stations, were estimated as the lowest for AL and the highest for SP in 2019, while they were the lowest for BW and the highest for Narela in 2020, and the lowest for PG and the highest for SP in 2021. 

For benzene, some monitoring stations had LCR values that exceeded the standard LCR value as prescribed by the CPCB, such as values of 1.06 × 10^−5^, 1.43 × 10^−5^, 2.07 × 10^−5^, 2.24 × 10^−5^, 2.25 × 10^−5^, 2.25 × 10^−5^, and 2.71 × 10^−5^ for PG, NL, WZ, SON, MD, OKH, and SP, respectively, in 2019; 1.26 × 10^−5^, 1.48 × 10^−5^, 1.57 × 10^−5^, 1.58 × 10^−5^, 2.32 × 10^−5^. 5.69 × 10^−5^, and 4.92 × 10^−4^ for MD, OKH, SON, WZ, SP, NG, and NL, respectively, in 2020; 1.06 × 10^−5^, 1.29 × 10^−5^, 1.49 × 10^−5^, 1.81 × 10^−5^, 1.98 × 10^−5^, 2.02 × 10^−5^, and 2.63 × 10^−5^ for AL, WZ, NL, SON, MD, OKH, and SP, respectively, in 2021. For the pre-lockdown period, a value of LCR was established to be similar to 2.15 × 10^−5^ and 2.05 × 10^−5^ for male and female residents, respectively, in China, which showed discernibly higher carcinogenic risks for male and female residents [61].

For children, the total LCR values for benzene at all of the monitoring stations were calculated to be 2.89 × 10^−5^, 1.07 × 10^−4^, and 2.41 × 10^−5^, whereas the values for toluene were calculated to be 3.05 × 10^−4^, 3.05 × 10^−4^, and 3.05 × 10^−4^, for 2019, 2020, and 2021, respectively. The LCR values for benzene for children ranged from 1.05 × 10^−6^ to 4.52 × 10^−5^, from 2.42 × 10^−6^ to 8.21 × 10^−4^, and from 4.59 × 10^−6^ to 4.39 × 10^−5^ in 2019, 2020, and 2021, respectively. The LCR values for benzene were estimated for all of the monitoring stations with the lowest LCR value for AL and the highest LCR value for SP in 2019, while in 2020, the lowest LCR value was for BW and the highest LCR value was for NL, and in 2021, the lowest LCR value was for PG and the highest LCR value was for SP. For benzene, some monitoring stations had LCR values that exceeded the standard LCR value as prescribed by the CPCB, such as in 2019; all stations exceeded the LCR value ranging from 1.05 × 10^−5^ for AL to 4.52 × 10^−5^ for SP; in 2020, the LCR values were 1.22 × 10^−5^, 2.10 × 10^−5^, 2.46 × 10^−5^, 2.61 × 10^−5^, 2.63 × 10^−5^, 3.86 × 10^−5^, 9.49 × 10^−5^, and 8.20 × 10^−4^ for AL, MD, OKH, SON, WZ, SP, NG, and NL, respectively; in 2021, the LCR values were 1.77 × 10^−5^, 2.16 × 10^−5^, 2.49 × 10^−5^, 3.01 × 10^−5^, 3.29 × 10^−5^, 3.36 × 10^−5^, and 4.39 × 10^−5^ for AL, WZ, NL, SON, MD, OKH, and SP, respectively. For all monitoring stations, the LCR values for benzene, for the pre-lockdown, lockdown, and post-lockdown periods, were higher than the authorized value (1 × 10^−6^), except during the lockdown period, which is a guideline limit value in some circumstances [62].

## 6. Conclusions

The quantifications of the selected volatile organic compounds (VOCs) were executed in various industrial areas in Delhi, India, from 2019 to 2021. The VOC data from 2019 to 2021 were acquired from the Central Pollution Control Board (CPCB) website, with reference to the pre-pandemic, pandemic, and post-pandemic periods. Using statistical analysis, the current study concluded that anthropogenic activities were considerable sources of emission for VOCs in industrial areas. At all monitoring stations, the mean VOC levels were 47.22 ± 30.15, 37.19 ± 37.19, and 32.81 ± 32.81 µg/m^3^ for 2019, 2020, and 2021, respectively. As a result, the level of TVOCs gradually deteriorated over consecutive years due to the pandemic. During the lockdown, the major factors behind the crucial decrease in TVOC levels were complete and partial restrictions on industrial activities, transport, and marketplace openings. The average TVOC values at all the monitoring stations declined from 9 to 61% throughout the pandemic period in contrast to the pre-pandemic period. The change in TVOC levels was reported to be the highest in NL, because NL is renowned for plastic manufacturing industries that create shoe soles and other additional plastic goods, such as adhesive, Rexine, and other tremendously explosive items, which could be significant sources of emissions during the reopening of industrial activities. During 2020, the T/B ratio was estimated in the range of 0.03–13.47, indicating that the major contributors were traffic and non-traffic sources, whereas, during 2021, it ranged from 3.54 to 42.10, where high ratios stipulated non-traffic sources and much higher ratios indicated industries, factories, and petrol pumps as the predominant contributors. The correlation results revealed that TVOC levels had negative relationships with wind speed and atmospheric temperature, which might play a significant role in the dispersion of TVOCs. Comparatively, the lifetime cancer risk (LCR) value for males and females was estimated to be higher throughout the lockdown period than in the pre- and post-lockdown periods. The reason could be the longer exposure time to increase the production of plastic and resin manufacturing units during the pandemic period. Further, the present study aims to increase the scientific accuracy of research on VOCs. 

## Figures and Tables

**Table 1:** Different monitoring stations with latitude, longitude, and population census at Delhi.

S. No.	Monitoring Stations	Latitude (0 E)	Longitude (0 N)	Population Density (2021 Census) (Km^−2^)
1.	Alipur (AL)	77.1331	28.7972	6369
2	Bawana (BW)	77.0483	28.7932	6660
3	Mundaka (MD)	77.0349	28.6823	10,275
4	Najafgarh (NG)	76.9855	28.6090	5213
5	Narela (NL)	77.0892	28.8549	3071
6	Okhala (OKH)	77.2914	28.5626	31,087
7	Patparganj (PG)	77.3046	28.6347	22,088
8	Shadipur (SP)	77.1582	28.6516	23,942
9	SoniaVihar (SON)	77.2496	28.7332	5662
10	Wazirpur (WA)	77.1604	28.6975	24,908

**Table 2:** Correlation between the different monitoring stations for the pre-pandemic period.

	AL	BW	MD	NG	NL	OKH	PG	SP	SON	WA
AL	1									
BW	0.003	1								
MW	0.306 **	0.306 **	1							
NG	0.234 **	0.302 **	0.734 **	1						
NL	−0.006	0.434 **	0.286 **	0.448 **	1					
OKH	0.062	0.153 **	0.296 **	0.351 **	0.178 **	1				
PG	−0.140 **	0.081	0.116 *	0.019	0.116 *	0.094	1			
SP	0.192 **	0.206 **	0.441 **	0.317 **	0.325 **	0.139 **	0.310 **	1		
SON	0.227 **	0.148 **	0.545 **	0.337 **	0.036	0.167 **	0.222 **	0.106 *	1	
WA	−0.081	0.044	−0.005	−0.207 **	0.022	−0.109 *	0.236 **	0.301 **	0.048	1

** Correlation is significant at the 0.01 level (2-tailed). * Correlation is significant at the 0.05 level (2-tailed).

**Table 3:** Correlation between the different monitoring stations during the pandemic period.

	AL	BW	MW	NG	NL	OKH	PG	SP	SON	WA
AL	1									
BW	0.255 **	1								
MW	0.300 **	0.360 **	1							
NG	0.343 **	0.440 **	0.575 **	1						
NL	0.454 **	0.499 **	0.413 **	0.665 **	1					
OKH	0.554 **	0.343 **	0.373 **	0.598 **	0.628 **	1				
PG	0.141 **	0.107 *	0.129 *	0.139 **	0.066	0.092	1			
SP	0.736 **	0.340 **	0.422 **	0.550 **	0.547 **	0.619 **	0.173 **	1		
SON	0.468 **	0.356 **	0.515 **	0.536 **	0.347 **	0.434 **	0.194 **	0.530 **	1	
WA	0.080	0.187 **	0.096	−0.013	0.077	0.023	−0.067	0.041	−0.038	1

** Correlation is significant at the 0.01 level (2-tailed). * Correlation is significant at the 0.05 level (2-tailed).

**Table 4:** Correlation between the different monitoring stations for the post-pandemic period.

	AL	BW	MW	NG	NL	OKH	PG	SP	SON	WA
AL	1									
BW	0.497 **	1								
MW	−0.021	−0.076	1							
NG	0.349 **	0.646 **	0.021	1						
NL	0.130 *	0.157 **	0.119 *	0.273 **	1					
OKH	0.219 **	0.176 **	0.197 **	0.309 **	0.571 **	1				
PG	0.240 **	−0.004	0.094	0.094	0.354 **	0.725 **	1			
SP	0.654 **	0.553 **	0.027	0.426 **	0.254 **	0.287 **	0.292 **	1		
SON	0.565 **	0.513 **	0.054	0.546 **	0.377 **	0.612 **	0.534 **	0.617 **	1	
WA	0.572 **	0.813 **	0.016	0.648 **	0.112 *	0.255 **	0.105 *	0.587 **	0.618 **	1

** Correlation is significant at the 0.01 level (2-tailed). * Correlation is significant at the 0.05 level (2-tailed).

**Table 5:** The correlation coefficient between TVOCs and meteorological parameters for the year 2019–2021.

Parameters	TVOCs	SR	BP	AT	RF	WS	WD
TVOCs	1						
SR	0.034	1					
BP	0.118	−0.176	1				
AT	−0.168	0.146	0.169	1			
RF	0.012	0.059	−0.992 **	−0.070	1		
WS	−0.150	0.123	0.077	−0.137	−0.047	1	
WD	0.007	−0.355	0.102	0.100	−0.061	−0.308	1

** Correlation is significant at the 0.01 level (2-tailed).

## Data Availability

Not applicable.
